# EDDA: An Efficient Distributed Data Replication Algorithm in VANETs

**DOI:** 10.3390/s18020547

**Published:** 2018-02-10

**Authors:** Junyu Zhu, Chuanhe Huang, Xiying Fan, Sipei Guo, Bin Fu

**Affiliations:** 1 School of computer, Wuhan University, Wuhan 430072, China; 2011@whu.edu.cn (J.Z.); wow@whu.edu.cn (X.F.); 2 Collaborative Innovation Center of Geospatial Technology, Wuhan University, Wuhan 430072, China; 3 School of Mathematics and Statistics, Central China Normal University, Wuhan 430079, China; gsp@mail.ccnu.edu.cn; 4 Department of Computer Science, The University of Texas Rio Grande Valley, Edinburg, TX 78541, USA; bin.fu@utrgv.edu

**Keywords:** VANETs, data dissemination, bounded number of messages, distributed consensus, sensor

## Abstract

Efficient data dissemination in vehicular ad hoc networks (VANETs) is a challenging issue due to the dynamic nature of the network. To improve the performance of data dissemination, we study distributed data replication algorithms in VANETs for exchanging information and computing in an arbitrarily-connected network of vehicle nodes. To achieve low dissemination delay and improve the network performance, we control the number of message copies that can be disseminated in the network and then propose an efficient distributed data replication algorithm (EDDA). The key idea is to let the data carrier distribute the data dissemination tasks to multiple nodes to speed up the dissemination process. We calculate the number of communication stages for the network to enter into a balanced status and show that the proposed distributed algorithm can converge to a consensus in a small number of communication stages. Most of the theoretical results described in this paper are to study the complexity of network convergence. The lower bound and upper bound are also provided in the analysis of the algorithm. Simulation results show that the proposed EDDA can efficiently disseminate messages to vehicles in a specific area with low dissemination delay and system overhead.

## 1. Introduction

Vehicular ad hoc networks (VANETs), which aim to improve transportation safety and enable data services for in-vehicle consumption, have attracted much interest [1]. By enabling vehicles to communicate with one another and creating a large network with vehicles acting as the network nodes, various types of information (e.g., traffic conditions, advertising news and e-coupons) can be shared among vehicles. Interconnected by means of vehicle-to-vehicle (V2V) communications, VANETs can exchange information about the states of vehicles and roads, as well as provide infotainment services [2,3]. Especially, with the considerable demands of sensing and transmitting data for services, the vehicles can be regarded as mobile sensors, which can sense real-time vehicular surroundings [4].

Data dissemination is a promising application in VANETs, where messages are carried and forwarded by vehicular sensor nodes cooperatively toward their destinations. Through V2V communication in VANETs, the safety-related and commercial contents can be efficiently disseminated among a large number of vehicular sensor nodes. Thus, VANETs have significant potential to enable diverse applications associated with transportation safety, traffic efficiency and infotainment. There are many data dissemination applications, such as the important example of VANET safety application, local danger warning (LDW). LDW creates a warning message for every detected hazard and informs the driver of its conditions [5]. In LDW applications, the information dissemination protocol has to implement strategies that provide reliable communication. There are also data dissemination applications and services that provide ad and infotainment information to users in VANETs, such as digital billboards [6], an ad service provider architecture for urban vehicular networks; an electric coupon system [7], which contains a bonus point-based coupon scheme and derives an optimal strategy that allows each user to determine how many bonus points she/he should ask for when passing the coupon; and FleaNet [8], a novel epidemic-based architecture that supports query dissemination, matching and notification routing and scales up to thousands of nodes without disrupting existing services. To fulfill the attractive applications by V2V data dissemination, messages can be disseminated via the vehicular environment. Extensive research works have been conducted to facilitate data dissemination, as it is of great importance in VANETs [9,10].

To achieve efficient data dissemination, data replication has been recognized as an effective approach for data delivery in vehicular networks [11]. Data replication enables multiple copies of the same data carried by different vehicles to be transmitted to a target destination simultaneously. Thus, the data will be distributed to a specific area in a quick manner. A variety of distributed algorithms has been proposed based on data replication [12]. Additionally, as VANETs are characterized by the lack of centralized control, distributed coordination among the nodes and averaging consensus problems are challenging issues in networks [13,14]. Due to the adaptability to dynamic network topologies, gossip algorithms, a distributed averaging approach based on rounds of communications between neighbor nodes, have received considerable attention because of their simplicity and robustness in noisy and uncertain environments [15]. The algorithms are usually compared by evaluating their speed of convergence, which can be measured by the number of transmissions. As gossip algorithms can waste significant energy by essentially passing around redundant information, significant efforts have been devoted to decrease the resource consumption, mainly through acceleration of the convergence rate. Similar to gossip algorithms, in replication-based data dissemination, each node possesses the message by utilizing a limited amount of local information to allow distributed knowledge of global network properties. In this research, we study distributed averaging to evaluate the efficiency of network convergence in replication-based data dissemination.

### 1.1. Our Goal

Dynamic data replication in distributed network systems can accelerate information spread in a specific area. However, possible issues with replication-based routing include: network congestion in clustered areas leading to a long delay, being wasteful of network resources (including bandwidth, storage and energy), and network scalability. Since network resources may quickly become constrained, deciding how to replicate the messages plays critical roles in many routing protocols. Meanwhile, some algorithms, such as epidemic and gossip algorithms, could cause significant network overhead by essentially passing around redundant information multiple times, which brings additional communication overhead.

On the other hand, by increasing the diversity of pairwise exchanges, data replication algorithms improve upon the convergence speed of data dissemination. As load balancing is an important goal in ad hoc networks, we let the data carrier distribute the data dissemination task to multiple vehicles in the area, and we hope every vehicle in the network can carry an approximately equal amount of data dissemination tasks. To achieve this, at each step, every vehicle computes a weighted average (mean of the two numbers) of its own value with values received from some of the other nodes until the network converges to a global average. The global average is calculated as the total number of message copies divided by the number of vehicles being averaged. The average calculation can be utilized to measure the convergence rate and complexity when network consensus is achieved in replication-based data dissemination. It is interesting to investigate how such nodes average their values, trying to achieve a general consensus in the shortest possible time.

To reduce unnecessary transmissions and improve communication overhead, the number of messages that can be replicated should be bounded when a message is disseminated to a specific area. Besides, through distributed averaging, the dissemination delay will decrease when the computing and communication burdens are distributed among the vehicles, which motivates us to combine distributed averaging with replication-based data dissemination. Therefore, we need to develop a data replication scheme that can control the number of message copies and combine distributed averaging with data dissemination to evaluate network convergence rate through data replication.

### 1.2. Main Contributions

Graph theory is used to help understand the topological properties of VANETs, where the vehicles and their communication links can be modeled as vertices and edges in the graph, respectively. According to the network traffic density, we divide the VANET topology into three types of graphs: linear graph, arbitrary graph and complete graph. In urban areas, due to fast speed of the vehicles, the network topology changes from time to time, so do the communication links. We can treat this type of network topology as arbitrary graph. In the scenario of a highway, assume vehicles move at a constant velocity along the road and every vehicle has full knowledge of its neighbors right next to it. In this situation, the network topology can be seen as a special case of arbitrary graph, that is a linear graph. We talk about the cases of arbitrary graph and linear graph in this study, while the case of complete graph will be discussed in the future work.

**Figure 1:**
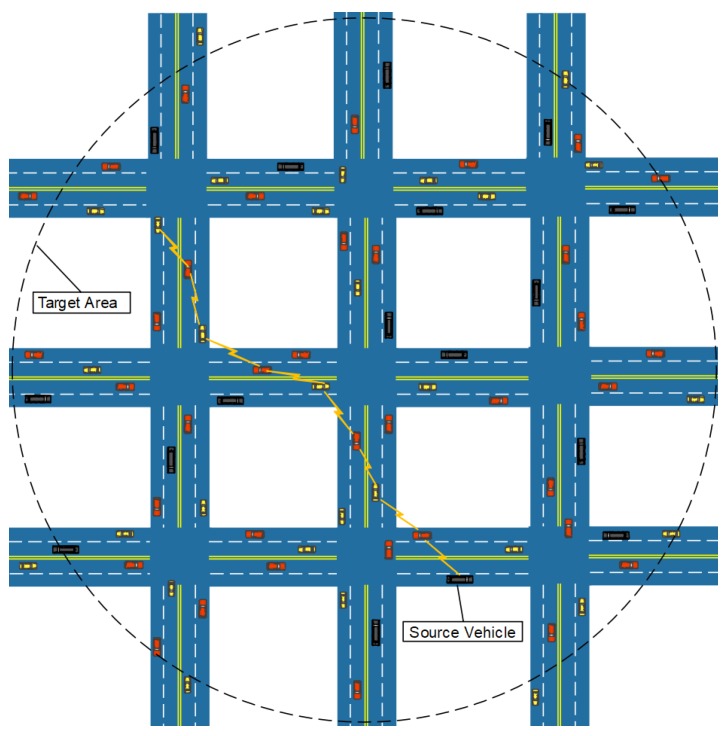
Data dissemination area.

This paper investigates the problem of a vehicle node disseminating a message to a large number of recipients in a specific area in VANETs, and the objective is to disseminate the message to the destination area with low dissemination delay and reduced communication overhead. As shown in Figure 1, a message is generated at the source node, carried and forwarded by the passing vehicles to vehicles in the destination region. During the message transmission process, the message can be forwarded from one vehicle to another by V2V communications. When two vehicle nodes meet, they will update their values of the corresponding message after an average operation until the network consensus is reached. When the network reaches a consensus, we say that it is ϵ-balanced. In this context, we propose a distributed data replication algorithm, EDDA, for arbitrary graph and linear graph.

Before more details are given, we give a description of the ϵ-balanced status to help understand the proposed idea. Assume each vehicle *i* in the region carries message *M* and a parameter $n_{i}\geq 1$, which indicates how many message copies *i* can spread. After average operations, if $n_{i}\left(i=1,\cdots ,m\right)$ carried by vehicle *i* satisfies the conditions $\sum_{i=1}^{m}n_{i}=n$ and $|n_{i}-n_{j}|\leq \epsilon$, we say that the network is ϵ-balanced. Here, *n* indicates the total number of copies, $n_{i}$ indicates the number of copies held by node *i* and ϵ∈(0,1].

To speed up the dissemination process, the proposed data replication algorithm lets the sender distribute the data dissemination task to vehicles in the same network by allowing each vehicle node to randomly choose one of their neighbors and average their current values, which indicate the amount of their assigned dissemination tasks. The pair of nodes computes the pairwise average, which then becomes the new value for both nodes. By iterating this pairwise averaging operations, it is easy to show that all the nodes converge to the global average in a completely distributed manner. Then, the paper analyzes the replication algorithm from the perspective of the approximation of how many nodes can receive the message when the system enters into a balanced status. It also presents the convergence speed of the network in the cases of arbitrary graph and linear graph. More specifically, we measure the complexity of network convergence by the number of communication stages in a distributed computing environment. A communication stage indicates an average operation among a set of independent edges in the connected graph. In each stage, each node is involved in at most one average operation. Theoretical analysis and simulation results show that the proposed algorithm can improve the efficiency of data dissemination and make the network converge to consensus quickly.

Besides, the dissemination delay and overhead can be measured respectively by network convergence speed and the number of communication stages needed by the network to be ϵ-balanced. Through cooperative communication of vehicle nodes, the data dissemination process will speed up; thus, the delay for the network to converge to a balanced status can be reduced. The number of communication stages is also reduced as the computing and communication burdens can be distributed among the nodes, which can be seen as a reflection of reduced communication overhead.

To summarize, the main contributions are described as follows.

(1) We apply graph theory to different scenarios in VANETs and divide the network into arbitrary graph, linear graph and complete graph. The cases of arbitrary graph and linear graph are discussed in this study. We propose a general system model for disseminating a bounded number of message copies in the network. Under this model, we develop EDDA, an efficient data replication algorithm that can be applied to both arbitrary graph and linear graph, in which the number of messages that can be replicated is limited and a network balanced status will be achieved.
(2) We derive the theoretical analysis to obtain the approximate number of nodes that would receive the message when the system achieves an ϵ-balanced status. The convergence speed of the algorithm is also presented. As a special case of arbitrary graph, detailed analysis of the upper bound and lower bound for linear graph is provided, to show the efficiency of the proposed algorithm. The effectiveness of our algorithm has been validated by extensive simulations.

### 1.3. Paper Organization

The rest of this paper is organized as follows. Section 2 overviews the related work on data replication and average consensus problems in vehicular ad hoc networks. Section 3 first describes the analytical model of data replication, then presents a data replication algorithm, which can be applied to arbitrary graph and linear graph, and finally analyzes the approximation property of the proposed algorithm. Detailed theoretical analysis of the algorithm on convergence speed of arbitrary graph and linear graph is given in the following Section 4. Section 5 presents the simulation environment and performance metrics to evaluate the performance of the compared algorithms. Finally, Section 6 concludes the paper.

## 2. Related Work

In this section, we give a brief overview of the related works. First, we discuss the research work on data dissemination in VANETs, then we introduce current research on the average consensus problem.

### 2.1. Data Dissemination Algorithms

Similar to other ad hoc networks, VANETs often suffer intermittent connectivity due to the high mobility of vehicles [16]. There are many existing works considering and studying how to develop efficient algorithms, to achieve low dissemination delay and system cost in VANETs [17,18,19]. As broadcast is the basic mechanism of VANET communication, flooding is the most common method in data dissemination. While flooding can achieve the maximum wireless coverage and rapid data dissemination, it may cause a serious broadcast storm. Torres et al. [20] compared ten different flooding-based algorithms and proposed an improved flooding scheme to cope with variant vehicle density situations. Yang et al. [21] first challenged the accuracy of the innovative assumption that was widely adopted in delay performance analysis of network-coding-based epidemic routing in delay-tolerant networks. Taherkhani et al. [22] proposed two strategies, DySch and TaSch. According to the strategies, the safety and service messages were assigned a priority value based on the characteristics of messages; such that the messages were disseminated dynamically and heuristically.

Although many technologies are proposed as potential solutions to support various communications in the network, V2V communication is still an important component of the VANET system. Related works using pure V2V communication are summarized as follows. Goonewardene et al. [23] designed a vehicle precedence algorithm to adaptively identify the nearby one-hop neighbors and select optimal cluster heads based on vehicle locations and velocities. Luo et al. [24] formed clusters based on geographically-divided grids, but they did not consider velocity and direction, which are important for accommodating the dynamic nature of VANETs. In [25], Ohta et al. used positions and moving direction of vehicles for clustering. Lin et al. [26] presented a novel moving-zone-based architecture and a corresponding routing protocol for message dissemination in VANETs by using pure V2V communications; the authors claimed that this was the first study that applied moving object techniques to vehicular networks. Kumer et al. [27] aimed to achieve lower message dissemination delay and reduce redundant rate. Bi et al. [28] used the time and angle method to determine the replicated node when RFID was not installed, to avoid network disconnection and improve message disseminating. Rivoirard et al. [29] proposed a clustering scheme that combines the information on the road configuration, vehicle mobility and link quality in order to build a structure relying only on the vehicles. Nilsson et al. [30] presented a measurement-based analysis of multilink shadowing effects in a V2V communication system with cars as blocking objects. Basheer et al. [31] reviewed several existing VANET safety applications and revised the disseminating methods for safety messages between vehicles without infrastructure to cover a wide area in a quick and reliable way.

In data dissemination, replication-based protocols have gained much attention in the scientific community, as they can allow for substantially better message delivery ratios than in forwarding-based protocols. These types of routing protocols allow for a message to be replicated; each of the replicas, as well as the original message itself are generally referred to as message copies or message replicas. Data replication-based protocols, such as epidemic routing [32], the PRoPHET routing protocol [33] and MaxProp [34], have been instrumented to intentionally minimize one of four metrics: average delay, missed deadlines, maximum delay and communication overhead.

Some algorithms controlled the replication rules in data dissemination. Spyropoulos et al. [35] proposed spray-and-wait (SW), a routing protocol that attempts to gain the delivery ratio benefits of replication-based routing, as well as the low resource utilization benefits of forwarding-based routing. Spray-and-wait can decrease the network resource consumption while it has similar performance compared to epidemic routing. However, it does not take into account bandwidth and network capacity. In contrast, Balasubramanian et al. [36] proposed a resource allocation protocol for intentional delay tolerant network (RAPID) , which decided whether to replicate packets whenever two vehicles encountered each other with respect to a given performance metric. RAPID explicitly calculated the effect of replication on the routing metric while considering resource constraints. To tackle the problems that data dissemination could not effectively address broadcast storm and network partition problems simultaneously, Akabane et al. [37] proposed a suitable urban multi-hop broadcast protocol (TURBO) that relied exclusively on local one-hop neighbor information to deliver messages under dense and sparse networks. Yan et al. [38] designed a data dissemination scheme, which can disseminate data to a desired number of receivers in VANET (DOVE). The scheme was inspired by processor scheduling treating roads as processors to optimize the workload assignment and improve the efficiency of on-road dissemination. Takahashi et al. [39] proposed an advanced routing scheme that controls the maximum number of replicas according to the distance between the source node and the nearest base station. They also showed how to decide the maximum number of replicas by analyzing the message delivery reliability of an existing DTN routing scheme. Li et al. [40] evaluated how many idle resources of LTE could be provided for safety services and how safety applications impacted LTE traditional users and proposed to reserve the idle radio resources in LTE for vehicular safety services. Additionally, the weighted-fair-queuing (WFQ) algorithm was proposed to schedule beacons for safety services using the LTE reserved resources. Li et al. [41] investigated the budget-constrained and delay-bounded roadside units’ (RSUs) placement problem in VANETs and formulated the budget constrained and delay-bounded placement (BCDP) problem to the budgeted maximum coverage problem, which was proven NP-hard. Heuristic algorithms were proposed to solve the problem, to reduce the RSU installation cost and provide a wide coverage for data dissemination. Chen et al. [42] investigated the data dissemination process in a cooperative communication mode. They fully utilized the capacity of VANETs’ mobility and then developed an analytical framework to model the capacity achieved by the vehicles of interest (VoIs) in VANET. Li et al. [43] proposed an adaptive quality of service (QoS)-based routing for VANETs (AQRV) , in which the intersections were adaptively chosen, through which data packets pass to reach the destination. To achieve the QoS constraints, the routing selection issue was mathematically formulated as a constrained optimization problem, and an ant colony optimization (ACO)-based algorithm was proposed to solve this problem. However, few of the schemes for data dissemination consider controlling the number of message copies in the network.

### 2.2. Average Consensus Problem

In cooperative control, a critical problem is to design appropriate protocols such that the group of agents can reach consensus on the shared information in the fixed and dynamically-changing interaction topologies. A consensus problem means convergence to a common value in the literature [44]. As a special case, an average consensus problem means convergence to the average of initial values, which is important in distributed decision-making for multiagent systems [45]. Average consensus is a fundamental problem in both distributed ad hoc networks and wireless sensor networks. The consensus status can be achieved through an iterative process of data transmission. During the past few decades, the problem attracted increasing interest in various research fields, and many gossip-based algorithms have been proposed to solve the consensus problem in wireless sensor networks [46,47].

Khosravi et al. [48] presented a gossip-based broadcast framework for strongly-connected topologies such as sensor networks, to solve the average consensus problem in an asynchronous environment where the network members might have different clock rates. Angelia et al. [49] first showed convergence to consensus under a bounded delay condition and some connectivity and intercommunication conditions imposed on the multi-agent system and then provided a bound on the time required to reach the consensus. Fabio and Sandro [50] investigated the consensus algorithms in large-scale networks; different from average preserving algorithms, they allowed it to reach consensus at a point that may be different from the average of the initial states. The advantage of such algorithms was that they did not need bidirectional communication among agents, and thus, they applied to more general contexts. To overcome the drawbacks of the standard packet-based gossip algorithms, Aysal et al. [51] studied a broadcast-based gossiping algorithm for wireless sensor networks and proved that the random consensus value was the average of initial node measurements and that it could be made arbitrarily close to this value in the mean squared error sense under a balanced connectivity model and by trading off convergence speed with the accuracy of the computation. Boyd et al. [52] analyzed the averaging problem under the gossip constraint for an arbitrary network graph and proposed a distributed subgradient algorithm that solves the optimization problem over the network. More importantly, the authors established a tight relation between the averaging time of the algorithm and the mixing time of an associated random walk, which could be utilized to design fast averaging algorithms for two popular networks: wireless sensor networks, modeled as geometric random graphs, and the Internet graph under the preferential connectivity (PC) model. Wu et al. [53] proposed and analyzed a family of broadcast gossip algorithms for strongly-connected directed graphs. If the network was symmetric (undirected) or if nodes knew their out-degree, these algorithms were guaranteed to converge to the average consensus both in expectation and in the mean-squared sense. Nedić et al. [54] assumed the underlying graph was the complete graph and that all the weights were equal; the convergence of the weighted-averaging dynamics was established only for double-stochastic weights. To improve the result of previous work in [54], the authors [55] investigated the properties of the weighted-averaging dynamic for the consensus problem and established convergence of the weighted-averaging algorithm for general time-varying graphs. Shi et al. [56] studied finite-time convergence of deterministic gossiping and showed that there existed a symmetric gossip algorithm that converged in finite time if and only if the number of network nodes was a power of two. They also proved that there always existed an asymmetric gossip algorithm with finite-time convergence for any number of nodes with asymmetric updates.

The aforementioned schemes for data dissemination and network consensus in vehicular networks together improve reliability, efficiency and persistency of network performance. However, few of them consider controlling the number of message that can be replicated in the network. Thus, the existing results from the known mechanisms cannot be applied to achieve the goals of this paper. This paper investigates the network consensus problem in a bounded number of message passing, which can be achieved through a number of average operations. Meanwhile, it mainly targets reducing redundant data transmission and alleviating network burden by distributing the communication and computing burden loads among vehicles. Toward this goal, we develop a mathematical framework for data dissemination and propose an efficient distributed data replication algorithm.

## 3. Bounded Number of Data Replication in Message Passing

In this section, we first give an example to explain how the bounded number of data replication works in Section 3.1. Section 3.2 presents definitions and models used in the bounded number of messages passing. Then, Section 3.3 proposes an efficient data replication algorithm to reduce dissemination delay and redundant transmissions. An approximation of the number of nodes that receive the message is derived in Section 3.4.

### 3.1. An Example

In this part, we will show that data replication and the bounded number of message copies are quite useful in accelerating the information spread in a specific area. On the one hand, they can improve upon convergence speed of the network and realize network equilibrium. Besides, when compared with the basic broadcast mechanism, giving a restriction on the number of message copies helps to reduce redundant data dissemination. To explain how the bounded number of data replication works, an example is given as below.

We consider three different scenarios of VANETs, urban environment, highway and parking lot, which respectively correspond to arbitrary graph, linear graph and complete graph. The way that the mechanism works in different scenarios is illustrated separately.

Case 1: Assume in the common urban environment that the traffic can be described as arbitrary graph. If a vehicle carries message *M*, it needs to spread the message to its neighborhood district. Assume the total number for which the message can be replicated is n=100 and the number of vehicles in this area is 20. The number of messages can be copied by each vehicle is denoted by $n_{i}$. We have $n_{1}=100$ and $n_{j}=0$ for j>1. Then, Vehicle 1 with $n_{1}=100$ communicates with a vehicle *j* within its communication area and $n_{j}=0$, and then, the message parameters of the two vehicles take the average, with $n_{1}=n_{j}=\frac{n_{1}+n_{j}}{2}=50$. The rest may be deduced by analogy. The ideal result is that each vehicle in this area carries message *M* with $n_{i}=5$. Until then, the network enters into a balanced status.

Case 2: Assume the message passing happens on the highway, where the traffic can be described as linear graph. We keep the same parameters. It takes less average operations than arbitrary graph for the network to enter into a balanced status.

Case 3: Assume the message passing happens in a parking lot. As there are many vehicles in the parking lot, we consider the traffic as a complete graph. Then, there might be many vehicles in one vehicle’s communication range. When the vehicle that carries message *M* with $n_{i}=100$ receives more than one communication request, it chooses the vehicle with $n_{j}$ if the gap between $n_{i}$ and $n_{j}$ is the largest. We hope that every vehicle in the neighborhood area receives message *M* and holds parameters with $|n_{i}-n_{j}|\leq \epsilon$, which means the network enters into a ϵ-balanced status and the network equilibrium is realized.

### 3.2. Definitions and Model

Assume vehicle *i* in the region carries message *M* and a parameter $n_{i}\geq 1$, where $n_{i}$ indicates the number of copies assigned to be spread by vehicle *i*. The total dissemination tasks of all the participant vehicles should meet the condition, which is $\sum_{i=1}^{m}n_{i}=n$. Here, *n* indicates the maximum number of copies that *M* can be replicated and spread in the network. If $n_{i},n_{j}\left(i,j\in 1,\cdots ,m\right)$ carried by any two vehicles *i* and *j* satisfy the condition $|n_{i}-n_{j}|\leq \epsilon$, we say the system is ϵ-balanced. An ϵ-balanced status will be obtained after a series of average operations. We define some concepts in this section.

When a node carries message *M* and it controls at most *a* copies of message *M* to be distributed in the network, it must have a≥1, and each node with a nonzero value is at least one.

We need to define the concept of potential (energy) in order to analyze the number of stages for the system entering into a balanced status and need the following lemma.

**Lemma** **1.** Assume that a,b,c, and d are real numbers with a+b=c+d. Then, we have (1) $\left(a^{2}+b^{2}\right)-\left(c^{2}+d^{2}\right)=2\left(b-d\right)\left(b-c\right)$ and (2) $\left(a^{2}+b^{2}\right)-\left(c^{2}+d^{2}\right)\geq 0$ if a≤c≤d≤b.

**Proof.**  By the condition of the lemma, we have b−d=c−a and b−c=d−a. Thus, $\left(a^{2}+b^{2}\right)-\left(c^{2}+d^{2}\right)=\left(b-d\right)\left(b+d\right)+\left(a-c\right)\left(a+c\right)=\left(b-d\right)\left(b+d-c-a\right)=2\left(b-d\right)\left(b-c\right)$. This proves (1). The second part of this lemma can be easily proven with (1). ☐

We will utilize graph theory to help understand the topological properties of VANETs. The vehicles and their communication links can be respectively treated as vertices and edges in the graph. The graph definition is described as below.

**Definition** **1.** *For a set of vehicles, their connected graph is an undirected graph G(V,E) such that each node represents a vehicle and an edge between two nodes indicates that the corresponding vehicles are within the distance of communication.*

- In urban areas, due to fast speed of the vehicles, the network topology changes from time to time, so do the communication links. We can formalize this type of network topology as arbitrary graph.
- In the scenario of a highway, assume vehicles move at a constant velocity along the road and every vehicle has full knowledge of its neighbors right next to it. In this situation, the network topology can be seen as a special case of arbitrary graph, that is linear graph.

**Definition** **2.** Let M be a message. Let G(V,E) be the connected graph for a set of vehicles. If each node i has a parameter $n_{i}$ to control the number of copies of message M that i can replicate, then G(V,E) associated with $n_{i}$ becomes a graph with a bounded number of message copies.

**Definition** **3.** *Let G(V,E) be a connected graph. Each node of G is assigned a nonnegative number $n_{i}$. The nodes of G are ϵ-balanced in the corresponding bounded message graph if the following conditions are satisfied:*

- Each node of G with $n_{i}>0$ satisfies $n_{i}\geq 1$.
- For every two nodes with $n_{i},n_{j}>0$, $|n_{i}-n_{j}|\leq \epsilon$, and
- There is no edge between nodes of values $n_{i}$ and $n_{j}$ in G, respectively, such that $n_{i}\geq 2$ and $n_{j}=0$.

**Definition** **4.** *Let R be the set of real numbers and N be the set of nonnegative integers. Define the following concepts:*

- A real average function A(.,.) is a mapping R×R→R×R, such that for two numbers a≤b, $A\left(a,b\right)=\left(\frac{a+b}{2},\frac{a+b}{2}\right)$ if a+b≥2, or A(a,b)=(a,b) if a+b<2.
- An integer average function A(.,.) is a mapping N×N→N×N such that for two numbers a≤b, A(a,b)=(k,k) if a+b=2k≥2, A(a,b)=(k,k+1) if a+b=2k+1≥2, or A(a,b)=(a,b) if a+b<2.
- For a list $L:a_{1},a_{2},\cdots ,a_{m}$ of numbers, define the potential of L to be $P\left(L\right)=a_{1}^{2}+a_{2}^{2}+\cdots +a_{m}^{2}$.
- For an average function A(.,.), define $S_{A}\left(\langle a,b\rangle \right)=2\left(b-d\right)\left(b-c\right)$, where A(a,b)=(c,d). Number b is considered a bar of length b. $S_{A}\left(\langle a,b\rangle \right)$ can be considered a small piece of length b−d from the bar of length b to go down by (b−c). Function $S_{A}\left(.\right)$ gives the potential change after an average operation (See Lemma 1).
- Let A(.,.) be an average function. Assume that $a_{1},a_{2},$$\cdots ,a_{n}$ is a list of numbers. It is transformed into another list $a_{1}^{\prime },a_{2}^{\prime },\cdots ,a_{n}^{\prime }$ by a series of average operations. Define its sum of the product to be $S\left(H\right)=\sum_{(a,b)\in H}S_{A}\left(a,b\right)=P\left(L\right)-P\left(L^{\prime }\right)$ (see Lemma 1), where H is the set of tuples (a,b) that take average operations to transform the first list into the second list. It is considered as the change of the potential after taking a series of average operations.

**Definition** **5.** A stage of communication is an average operation among a set of independent edges in the connected graph, which consists of a set of nodes. Two nodes connected by one of the independent edges can communicate. It allows those pairs of nodes to exchange messages in parallel.

**Definition** **6.** Convergence speed represents the speed that the network can enter into an ϵ-balanced status. In this paper, we use the number of stages (see Definition 5) to reflect the convergence speed.

**Figure 2:**
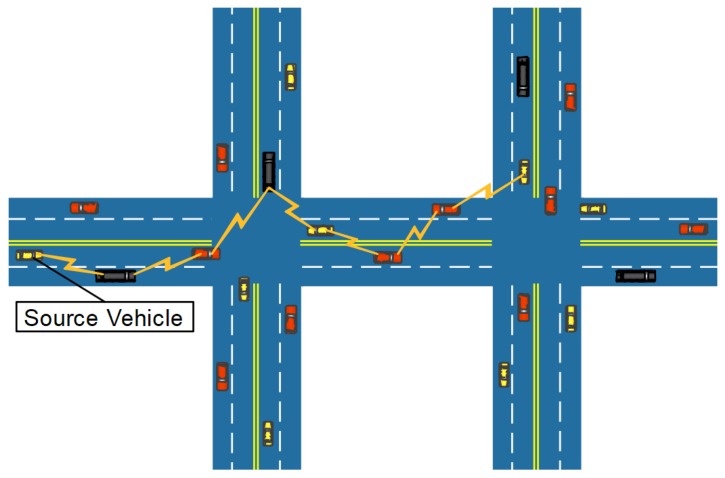
Task assignment process.

We use the number of stages to characterize the complexity to enter into a balanced status among a set of nodes whose communication is based on their connected graph, in which each node represents a vehicle and every edge connects two vehicles within a distance no greater than their range of communication. Furthermore, the number of stages can reflect how many transmissions are needed to achieve an ϵ-balanced status. Thus, the number of stages can be utilized to denote communication overhead in the network. Through the stages, the message dissemination tasks are distributed to the number of nodes for cooperative delivery. The process of task assignment is as shown in Figure 2.

### 3.3. Algorithm

Here, to improve the performance of data dissemination, we propose an efficient data replication algorithm EDDA that can make the network system enter an ϵ-balanced status in a short time, which is presented as algorithm 1.

In the proposed data replication algorithm, a message *M* is carried by one specific node. We need to distribute the message to the nodes in a given area. The total number of messages *M* that can be replicated is controlled by a parameter *n*. Each node *i* receives a corresponding parameter $n_{i}\geq 1$ to control the number of copies for which *M* can be replicated when it receives message *M*, where $n_{i}$ indicates the number of copies that can be distributed by node *i*. After a certain number of stages, those $n_{1},\cdots ,n_{m}$ will enter into a balanced status (see Definition 3). The complexity to enter into a balanced status is measured by the number of stages that are counted in algorithm 1. To give an overview, EDDA schedules with the following three steps.

(1) First, EDDA constructs the graph and initializes the value of every vertex of graph *G*. In the initialization procedure (see algorithm 2), the vehicle node that carries the message will be assigned a value of *n*. All other nodes will be assigned a value of zero, which means they do not have the message.
(2) Second, select independent edges from *G* so different pairs of nodes can communicate with each other in parallel. After the selection, replace the values of nodes with their new values by taking the average of the current values. Each stage should update the values of the nodes in the graph one time. Then, go to the next stage, and stop the average operations until the system is ϵ-balanced.
(3) Third, EDDA outputs graph $G^{\prime }$, with the final values of all nodes updated. If new nodes enter into the network and break the balance, the procedures will be executed again to achieve network balance.

The pseudocode of EDDA is described as follows, the initialization procedure is presented separately as algorithm 2.

### 3.4. Approximation

To analyze the proposed algorithm, we discuss how many nodes can receive the message when the system achieves an ϵ-balanced status.

**Lemma** **2.** Assume that L is a list of numbers. Let H be a finite set of tuples taking average operations when the list is transformed into list $L^{\prime }$. Then, the sum of numbers in $L^{\prime }$ is the same as the sum of numbers in L.

**Algorithm 1** Data replication algorithm.**Input:** bounded message graph *G* (see Definition 2); parameter ϵ **Output:** Bounded message graph $G^{\prime }$; number of stages *a* 1: Call Algorithm 2; 2: Let a=0; 3: **repeat** 4:  Select independent edges (disjoint pairs of *G*) $\left(i_{1},j_{1}\right),\left(i_{2},j_{2}\right),\cdots ,\left(i_{k},j_{k}\right)$ with $n_{i_{t}}\neq n_{j_{t}}$ for t=1,⋯,k; 5:  **for**
t=1 to *k*
**do** 6:   $n_{i_{t}}=\left(n_{i_{t}}+n_{j_{t}}\right)/2$; 7:   $n_{j_{t}}=\left(n_{i_{t}}+n_{j_{t}}\right)/2$; 8:  a=a+1; 9:  let $n_{max}$ and $n_{min}$ be the maximum and minimum value of *G*, respectively; 10: **until**
$(n_{max}-n_{min}\leq \epsilon$) 11:  **return**
*a* and graph $G^{\prime }$ with updated values;

**Algorithm 2** Initialization.**Input:** Graph *G*; parameter *n* **Output:** Weighted graph *G* with values 1: Let $v_{i}$ denote the nodes in graph *G*; 2: Let $v_{1}$ (i=1) denote the node that carries message *M*; 3: Let $n_{v_{i}}$ denote the assigned value of node $v_{i}$; 4: $n_{v_{1}}=n$; 5: **for**
i=2 to *m*
**do** 6:  $n_{v_{i}}=0$;

**Proof.**  It follows from the definition of the average operation. ☐

**Theorem** **1.** Let m be the number of nodes that form a connected graph and n be an integer to control the number of copies of the message to be sent. Then, when the system enters into an ϵ-balanced status, there are at least $\min(\frac{n}{2+\epsilon },m)$ nodes that have received the message.

**Proof.**  When the system is ϵ-balanced, we have $|n_{i}-n_{j}|\leq \epsilon$ for any two nodes that can communicate. We also have $n_{i}\geq 1$ for each $n_{i}\neq 0$.

Case 1. Every $n_{i}\neq 0$. In this case, we have $n_{i}\geq 1$, and all *m* nodes have received the message. It is trivial to see $m\geq \min(\frac{n}{2+\epsilon },m)$.

Case 2. There is at least one $n_{i}$ with $n_{i}=0$. In this case, each positive $n_{i}$ satisfies $n_{i}\leq 2+\epsilon$. Otherwise, there is a pair of neighbors $n_{s}$ and $n_{t}$ such that $n_{s}=0$ and $n_{t}\geq 2$ since *m* nodes are connected. They can take average operations and bring a contradiction. Therefore, the number of nodes that have received the message is at least $\frac{n}{2+\epsilon }$ by Lemma 2. ☐

## 4. Speed of Convergence on Arbitrary Graph and Linear Graph

In this section, we show that the system will converge to an ϵ-balanced status after a finite number of stages. We first derive the convergence speed of arbitrary graph, then present detailed analysis of the upper bound and lower bound of linear graph.

### 4.1. Arbitrary Graph

In this part, we discuss the convergence speed of arbitrary graph. Lemma 1 shows that the potential of a list goes down after each average operation.

**Definition** **7.** *Let real number w≥0. Let G(V,E) be a graph and $V^{\prime }\subseteq V$. If every node $v\in V^{\prime }$ has a weight at least w, then $V^{\prime }$ is a w-region of G. A w-spread of G satisfies the conditions:*

*1.*  A node with a weight of at least 2w can take the average with a node with weight zero, and
*2.*  two nodes with a weight of at least w can take the average.

**Lemma** **3.** Assume that every node in the list of nodes in an arbitrary graph G(V,E) has a weight of at least w. A w-spread takes at most $\frac{4n^{4}}{\epsilon }$ stages of real average operations to reach the ϵ-balanced state.

**Proof.**  Let *L* be the list of nodes with weights in the beginning. Clearly, we have $P\left(L\right)\leq n^{2}$.

Assume that there are two nodes with $n_{u}-n_{v}>\epsilon$. There is a path from *u* to *v* in the graph such that each node has a value of at least one. Thus, the path length has at most *n* nodes. There are two nodes with values $n_{i}$ and $n_{j}$ such that $n_{j}-n_{i}\geq \frac{\epsilon }{n}$. Thus, $S_{A}\left(n_{i},n_{j}\right)\geq {\left(\frac{\epsilon }{2n}\right)}^{2}$.

Therefore, the total number of stages is at most $\frac{P(L)}{{\left(\frac{\epsilon }{2n}\right)}^{2}}\leq \frac{n^{2}}{{\left(\frac{\epsilon }{2n}\right)}^{2}}=\frac{4n^{4}}{\epsilon^{2}}$. ☐

**Theorem** **2.** Let w be greater than zero. Assume that the list of nodes in an arbitrary graph G(V,E) has a total sum of weights at most n. It takes at most $\frac{4n^{4}\cdot \min\left(m,\frac{n^{2}}{w^{2}}\right)}{\epsilon }$ stages of w-spread operations to reach the ϵ-balanced state, where m is the number of nodes in the graph.

**Proof.**  Let *L* be the list of nodes with weights in the beginning. We have $P\left(L\right)=n^{2}$. The number of average operations between a node with $\left(n_{i}\geq 2w\right)\wedge \left(n_{i}>0\right)$ and a node with $n_{j}=0$ is at most *m*. The number of the average number of average operations between a node with $\left(n_{i}\geq 2w\right)\wedge \left(n_{i}>0\right)$ and a node with $n_{j}=0$ is at most $\frac{n^{2}}{w^{2}}$ since an average operation has $S\left(n_{i},n_{j}\right)\geq w^{2}$. This follows from Lemma 3. ☐

### 4.2. Upper Bound for Linear Graph

Now, we discuss the convergence speed of linear graph that generally describes the traffic on a highway. We believe linear graph is an important special case that should be studied.

**Definition** **8.** A lineargraphG(V,E) consists a list of nodes $v_{1},v_{2},\cdots ,v_{n}$, and the set of edges $E=\{v_{1}v_{2},v_{2}v_{3},$$\cdots ,v_{i}v_{i+1},\cdots ,v_{n-1}v_{n}\}$. In other words, each edge connects two consecutive nodes in the list $v_{1},v_{2},\cdots ,v_{n}$, and every two consecutive nodes has an edge connecting them.

**Theorem** **3.** Assume that the list of nodes in a linear graph G(V,E) starts with the initial values L:n,0,0,⋯,0. Then, it takes at most $O(\frac{n^{2}}{\epsilon })$ stages of real average operations to reach the stage of ϵ-balance for a linear graph.

**Proof.**  The total number of average operations $A(n_{i},0)$ with $n_{i}\geq 2$ is at most *n*. We focus on the number of average operations $A(n_{i},n_{i+1})$ with $n_{i},n_{i+1}\geq 1$.

Let $n_{1}\geq n_{2}\geq n_{3}\cdots \geq n_{m}$ with m≤n since there are at most *m* nodes with a value of at least one. Let $d_{i}=n_{i}-n_{i+1}$.

Let each node *i* take an average operation with one of the neighbors j∈{i−1,i+1} such that *i* and *j* do not take the average in the last stage. In two consecutive stages, we have made at least contribution $\sum_{i=1}^{m-1}{\left(\frac{d_{i}}{2}\right)}^{2}\geq \frac{{\left(\sum_{i=1}^{m-1}d_{i}/2\right)}^{2}}{m-1}=\frac{{\left(\sum_{i=1}^{m-1}d_{i}\right)}^{2}}{4(m-1)}$ to S(H) by the Cauchy–Schwarz inequality, where *H* is the set of all average operations.

Let *k* be the minimum integer with $\frac{n}{2^{k}}\leq \epsilon$. Divide $\left[\frac{n}{2^{k}},n\right]$ into O(logn) intervals: $I_{1}=\left(\frac{n}{2},n\right],I_{2}=(\frac{n}{2^{2}},$ $\frac{n}{2}],\cdots ,I_{k}=\left(\frac{n}{2^{k}},\frac{n}{2^{k-1}}\right]$. The region *t* represents the interval $I_{t}$ such that $n_{1}-n_{m}\in \left(\frac{n}{2^{t+1}},\frac{n}{2^{t}}\right]$. When the system enters into the balanced status, we have $n_{1}-n_{m}\leq \frac{n}{2^{t}}\leq \epsilon$. To achieve the maximum potential, we assume there are $2^{t}$ numbers, which are equal to $\frac{n}{2^{t}}$ (which satisfies $2^{t}\cdot \frac{n}{2^{t}}\geq n$). According to the definition of potential $P\left(L\right)=a_{1}^{2}+a_{2}^{2}+\cdots +a_{m}^{2}$, here $a_{1}=\cdots =a_{m}=\frac{n}{2^{t}}$, $m=2^{t}$, we can get that the maximum potential of a list in region *t* is at most $2^{t}{\left(\frac{n}{2^{t}}\right)}^{2}$ (see Part 2 of Lemma 1). Therefore, it takes at most $2^{t}{\left(\frac{n}{2^{t}}\right)}^{2}\cdot \frac{1}{\frac{{\left(\frac{n}{2^{t}}\right)}^{2}}{4(m-1)}}\leq 4\left(m-1\right)2^{t}$ stages in the region *t*.

The total number of stages is bounded by $n+\sum_{t=1}^{k}4m2^{t}=O\left(\frac{n^{2}}{\epsilon }\right)$. ☐

**Theorem** **4.** Let w be a parameter greater than zero. Assume that the list of nodes in a linear graph G(V,E) starts with the initial values L:n,0,0,⋯,0. Then, it takes at most $O(\frac{n^{2}}{\epsilon }\cdot \min\left(m,\frac{n}{w}\right))$ stages of w-spread operations to reach the stage of ϵ-balance for a linear graph.

**Proof.**  Since the total number of nodes is *m*, there are at most *m* times that the average operation involves a node with zero value. There are at most $\frac{n}{w}$ nodes to have a value of at least *w*. This follows from Theorem 3. ☐

According to the above theorems, the list of nodes in a linear graph G(V,E) with initial values L:n,0,0,⋯,0 reaches the balance after certain stages. Assume a new node with value zero joins the list; it is obvious that the balance will be broken. As the values of the new list are smaller than the initial ones, it should take fewer real average operations for the system to enter into a new balanced status, which is relatively quicker than the previous balance process.

### 4.3. Lower Bound for Linear Graph

Following the result of the upper bound, we derive a nontrivial super linear lower bound for linear graph. It is derived based on some bottleneck properties for messages passing over a linear graph.

We consider the linear graph for which Node 1 connects to Node 2, node *i* connects to both node i−1 and node i+1 for 1<i<m and node *m* connects to node m−1. For $m=\frac{n}{8}$, it is trivial to see a linear lower bound to enter into ϵ-balance since it takes Ω(m) stages for a positive value to reach node *m* from Node 1 in a linear graph.

**Definition** **9.** Assume that, Node 1 has value n, and all of the other nodes have zero in the beginning. A normalized process is that at stage j with odd j, node 2i−1 takes the average with 2i for i=1,2,⋯; and, at stage j with even j, node 2i takes the average with 2i+1 for i=1,2,⋯.

**Definition** **10.** Assume that m nodes have values $n_{1}\geq n_{2}\geq \cdots \geq n_{m}$, respectively. The gap at node i is defined by $n_{i}-n_{i+1}$.

For a node *i* with a value of nine and its neighbor node i+1 with a value of three, both nodes have a value of $\frac{9+3}{2}=6$ after taking the average operation. Node *i* sends the 9−6=3 value to node i+1, and node i+1 receives three from this average operation. Each node can only take one average operation each moment. Average operations do not change the total values among the nodes.

**Definition** **11.** For each node i, define Rec(i,j) to be the amount that node i has received after j stages and Snd(i,j) to be the amount that node i has sent after j stages.

**Definition** **12.** For each node i, define MaxRec(i,j) to be the largest amount that node i can receive after j stages and MaxSnd(i,j) to be the amount that node i can send after j stages. Define Current(i,j) to be the current value at node i right after stage j.

**Lemma** **4.** Let $g_{1,j},g_{2,j},\cdots ,g_{m^{\prime },j}$ be the positive gaps (see Definition 10) between two consecutive nodes in the linear graph from Node 1 to node m after stage j. Using normalized processes, we always get $g_{1,j}\geq g_{2,j}\geq \cdots \geq g_{m^{\prime },j}$.

**Proof.**  This follows a simple induction. It is trivial at Stage 0 since we only have Node 1 with a value of *n*, and all of the other nodes have a value of zero after Stage 0. Assume that this is true after stage *j*. Consider stage j+1. After stage j+1 in the normalized process, every positive gap after stage j+1 is equal to $\frac{g_{i,j}+g_{i+1,j}}{2}$. For two consecutive gaps $\frac{g_{i,j}+g_{i+1,j}}{2}$ and $\frac{g_{i+1,j}+g_{i+2,j}}{2}$, we have $\frac{g_{i,j}+g_{i+1,j}}{2}\geq \frac{g_{i+1,j}+g_{i+2,j}}{2}$ because $g_{i,j}\geq g_{i+2,j}$ by the inductive hypothesis. Therefore, it is nondecreasing. ☐

**Lemma** **5.** Using the normalized stages, each node i has Rec(i,j)=MaxRec(i,j) and Snd(i,j)=MaxSnd(i,j) for all j.

**Proof.**  We prove this by contradiction. Assume that Rec(i,j)<MaxRec(i,j) or Snd(i,j)<MaxSnd(i,j) for some i,j. Let (i,j) be the the tuple such that it satisfies the following conditions:

- Either Rec(i,j)<MaxRec(i,j) or Snd(i,j)<MaxSnd(i,j),
- Integer *j* is the least, and (Rec(i,j)<MaxRec(i,j) or Snd(i,j)<MaxSnd(i,j)).

(A) We assume Rec(i,j)<MaxRec(i,j). Let $P^{*}$ be another process that has ${\mathrm{Rec}}^{*}\left(.\right),{\mathrm{Current}}^{*}\left(.\right),{\mathrm{Snd}}^{*}\left(.\right)$, and it has Rec∗(i,j)=MaxRec(i,j).

Case 1. i=1. It is trivial that Rec(i,j)=MaxRec(i,j)=n for all *j*. Therefore, we have a contradiction.

Case 2. i>1 and node *i* take the average with node i−1 at stage *j* in the normalized process. Node *i* receives flow from i−1; node i−1 has Rec(i−1,j−1)=MaxRec(i−1,j−1); and node *i* has Snd(i,j−1)=MaxSnd(i,j−1). Thus, we have $\mathrm{Current}\left(i,j\right)=\frac{\mathrm{MaxRec}(i-1,j-1)-\mathrm{MaxSnd}(i,j-1)}{2}$.

In this case, we have Current(i−1,j−1)>Current(i,j−1), and there is an average operation between node i−1 and node *i* at stage *j*.

We have:

$$\begin{array}{cc} & \mathrm{Current}(i-1,j)=\mathrm{Current}(i,j)\\ = & \frac{\mathrm{Current}(i-1,j-1)+\mathrm{Current}(i,j-1)}{2}\\ = & \frac{\left(\mathrm{MaxRec}(i-1,j-1)-\mathrm{MaxSnd}(i-1,j-1)\right)}{2}\\ & +\frac{\left(\mathrm{MaxRec}(i,j-1)-\mathrm{MaxSnd}(i,j-1)\right)}{2}\\ = & \frac{\mathrm{MaxRec}(i-1,j-1)-\mathrm{MaxSnd}(i,j-1)}{2}. \end{array}$$

Thus, we have $\mathrm{Current}\left(i-1,j\right)=\mathrm{Current}\left(i,j\right)=\frac{\mathrm{MaxRec}(i-1,j-1)-\mathrm{MaxSnd}(i,j-1)}{2}$.

Thus,

$$\begin{array}{cc} & \mathrm{Rec}(i,j)\\ = & \mathrm{Current}(i,j)+\mathrm{Snd}(i,j-1)\\ = & \frac{\mathrm{MaxRec}(i-1,j-1)-\mathrm{MaxSnd}(i,j-1)}{2}\\ & +\mathrm{MaxSnd}(i,j-1)\\ = & \frac{\mathrm{MaxRec}(i-1,j-1)+\mathrm{MaxSnd}(i,j-1)}{2}. \end{array}$$

Now, consider the process $P^{*}$. We have:

$$\begin{array}{cc} & {\mathrm{Rec}}^{*}\left(i,j\right)\\ = & {\mathrm{Current}}^{*}\left(i,j\right)+{\mathrm{Snd}}^{*}\left(i,j-1\right)\\ = & \frac{\left({\mathrm{Rec}}^{*}\left(i-1,j-1\right)-{\mathrm{Snd}}^{*}\left(i-1,j-1\right)\right)}{2}\\ & +\frac{\left({\mathrm{Rec}}^{*}\left(i,j-1\right)-{\mathrm{Snd}}^{*}\left(i,j-1\right)\right)}{2}\\ & +{\mathrm{Snd}}^{*}\left(i,j-1\right)\\ = & \frac{{\mathrm{Rec}}^{*}\left(i-1,j-1\right)+{\mathrm{Snd}}^{*}\left(i,j-1\right)}{2}. \end{array}$$

On the other hand, we have ${\mathrm{Rec}}^{*}\left(i-1,j-1\right)\leq \mathrm{MaxRec}\left(i-1,j-1\right)$ and ${\mathrm{Snd}}^{*}\left(i,j-1\right)\leq \mathrm{MaxSnd}\left(i-1,j-1\right)$. Thus, ${\mathrm{Rec}}^{*}\left(i,j\right)\leq \mathrm{Rec}\left(i,j\right)$. A contradiction.

Thus, Rec(i,j)=MaxRec(i,j). It is easy to see that MaxSnd(i−1,j)=MaxRec(i,j).

Case 3. i>1, and node *i* does not take the average with node i−1 at stage *j* in the normalized process.

We have Current(i−1,j−1)=Current(i,j−1) since there is no average operation between node i−1 and node *i*.

There is an average operation between node *i* and node i+1.

In this case, we have Rec(i,j)=Rec(i,j−1)=MaxRec(i,j−1) by the inductive hypothesis.

Now, consider process $P^{*}$. If node *i* does not take the average with node i−1 at stage *j* in $P^{*}$, by the inductive hypothesis, $\mathrm{Rec}\left(i,j\right)=\mathrm{MaxRec}\left(i,j-1\right)\geq {\mathrm{Rec}}^{*}\left(i,j-1\right)={\mathrm{Rec}}^{*}\left(i,j\right)$. A contradiction.

If node *i* does take the average with node i−1 at stage *j*, we still have that ${\mathrm{Rec}}^{*}\left(i,j-1\right)={\mathrm{Rec}}^{*}\left(i,j-2\right)$. Clearly, node *i* does not take the average with node i−1 at stage j−1 (otherwise, node *i* does not take the average with node i−1 at stage *j* since their values after stage j−1 are equal (Current(i−1,j−1)=Current(i,j))).

At the normalized process, node *i* takes the average with node i−1 at stage j−1. Thus, Rec(i−1,j−1)≤MaxRec(i−1,j−2) and Snd(i,j−1)≤MaxSnd(i,j−2).

We have:

$$\begin{array}{cc} & {\mathrm{Rec}}^{*}\left(i,j\right)\\ = & {\mathrm{Current}}^{*}\left(i,j\right)+{\mathrm{Snd}}^{*}\left(i,j-1\right)\\ = & \frac{\left({\mathrm{Rec}}^{*}\left(i-1,j-1\right)-{\mathrm{Snd}}^{*}\left(i-1,j-1\right)\right)}{2}\\ & +\frac{\left({\mathrm{Rec}}^{*}\left(i,j-1\right)-{\mathrm{Snd}}^{*}\left(i,j-1\right)\right)}{2}\\ & +{\mathrm{Snd}}^{*}\left(i,j-1\right)\\ = & \frac{{\mathrm{Rec}}^{*}\left(i-1,j-1\right)+{\mathrm{Snd}}^{*}\left(i,j-1\right)}{2}\\ \leq & \frac{\mathrm{MaxRec}(i-1,j-1)+\mathrm{MaxSnd}(i,j-1)}{2}\\ = & \frac{\mathrm{MaxRec}(i-1,j-2)+\mathrm{MaxSnd}(i,j-2)}{2}. \end{array}$$

On the other hand, node *i* takes the average with node i−1 at stage j−1. Thus,

$$\begin{array}{cc} & \mathrm{Rec}(i,j-1)\\ = & \mathrm{Current}(i,j-1)+\mathrm{Snd}(i,j-1)\\ = & \frac{\left(\mathrm{Rec}(i-1,j-1)-\mathrm{Snd}(i-1,j-1)\right)}{2}\\ & +\frac{\left(\mathrm{Rec}(i,j-1)-\mathrm{Snd}(i,j-1)\right)}{2}\\ & +\mathrm{Snd}(i,j-1)\\ = & \frac{\mathrm{Rec}(i-1,j-1)+\mathrm{Snd}(i,j-1)}{2}\\ = & \frac{\mathrm{Rec}(i-1,j-2)+\mathrm{Snd}(i,j-2)}{2}\\ = & \frac{\mathrm{MaxRec}\left(i-1,j-2\right)+{\mathrm{MaxSnd}}^{*}\left(i,j-2\right)}{2}. \end{array}$$

Thus, $\mathrm{Rec}\left(i,j-1\right)\geq {\mathrm{Rec}}^{*}\left(i,j\right)$. A contradiction.

(B) We assume Snd(i,j)<MaxSnd(i,j).

Since Snd(i,j)=Rec(i+1,j) and MaxSnd(i,j)=MaxRec(i+1,j), it transforms into the problem Rec(i+1,j)<MaxRec(i+1,j), which is Case A, and a contradiction has been derived. ☐

**Lemma** **6.** *For any constant δ>0, after $n^{\delta }$ normalized stages, Node* 1 *has a value of at most $\frac{n}{\sqrt{c\log n}}$ for some fixed c>0.*

**Proof.**  Let $f\left(n\right)=\sqrt{c\log n}$, where *c* will be determined later. After *k* stages, let $n_{1}\geq n_{2}\geq n_{3}\cdots \geq n_{k^{\prime }}$ be all values of nodes greater than zero. Clearly, $k^{\prime }\leq k$ since each stage can only make one node have a value of zero for a value greater than zero in the linear graph. Let $d_{i}=n_{i}-n_{i+1}$. Assume that $\sum_{i=1}^{k}d_{i}\geq \frac{n}{cf(n)}$.

Let each node *i* take an average operation with one of the neighbors j∈{i−1,i+1} such that *i* and *j* do not take the average in the last stage. In two consecutive normalized stages k−1 and *k*, the potential of the nodes is reduced by at least $\sum_{i=1}^{k}{\left(\frac{d_{i}}{2}\right)}^{2}\geq \frac{{\left(\sum_{i=1}^{k}d_{i}/2\right)}^{2}}{k}\geq \frac{n^{2}}{4kf{\left(n\right)}^{2}}$ by the Cauchy–Schwarz inequality.

After $h=n^{\delta }$ stages, the potential is reduced by at least $\sum_{k=1}^{h}\frac{n^{2}}{4kf{\left(n\right)}^{2}}\geq \frac{n^{2}}{4f{\left(n\right)}^{2}}\cdot \left(\sum_{k=1}^{h}\frac{1}{k}\right)\geq \frac{n^{2}\log h}{4df{\left(n\right)}^{2}}$ for some fixed positive real *d* since $\sum_{k=1}^{h}\frac{1}{k}=\Phi \left(\log h\right)$. Therefore, for $h=n^{\delta }$, $\frac{\log h}{4df{\left(n\right)}^{2}}\geq 1$ by selecting a fixed *c* to be small enough. Since the maximum potential is at most $n^{2}$, which is the beginning of the system for $n_{1}=n$ and $n_{i}=0$ for all i>1, after at most $h=n^{\delta }$ normalized stages, the value at Node 1 is $n_{1}=\sum_{i=1}^{k}d_{i}<\frac{n}{f(n)}$. ☐

**Theorem** **5.** It requires $\Omega (\max\left(n,m\right)\sqrt{\log\max(n,m)})$ stages to enter an ϵ-balance in a linear graph model.

**Proof.**  Assume that Node 1 starts with the largest value *n*. Let m=n/8. Let $f\left(n\right)=\sqrt{c\log n}$, where *c* is the same as that in Lemma 6. By Lemma 6, after $n^{\delta }<m/2$ normalized stages, we have $n_{1}\leq \frac{n}{f(n)}$. We still have at least m/2 nodes with zero values. We can assume that all stages are normalized stages by Lemma 5. This is because the normalized stages give the maximum flow from Node 1 to others. By Lemma 4, $d_{1}\geq d_{2}\geq \cdots$. In the rest of the stages, each stage sends at most $\frac{n_{1}}{m/2}\leq \frac{16}{f(n)}$ values to the area that contains the last $\frac{m}{2}$ nodes with zero values. When the system enters ϵ-balance for ϵ<1, each node must have a value of at least four. Thus, the total number of stages is at least $4\cdot \frac{m}{2}\cdot \frac{1}{\frac{16}{f(n)}}=\frac{1}{8}\cdot mf\left(n\right)=\Omega \left(n\sqrt{\log n}\right)=\Omega \left(\max\left(n,m\right)\sqrt{\log\max(n,m)}\right)$. ☐

## 5. Performance Evaluation

In this section, we first introduce the simulation environment, then present performance metrics and finally give a demonstration of the simulation results.

### 5.1. Simulation Setup

To evaluate the performance of the proposed replication algorithm, we have conducted extensive simulations. In the simulations, the following default settings are used.

**Figure 3:**
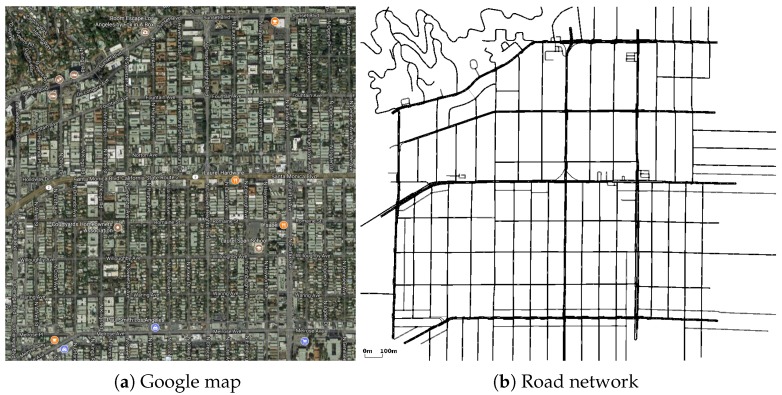
Los Angeles.

Compromised by the complexity of simulations, we select a bounded area on the map of Los Angeles for the simulations. We extract a 2000 m × 2000 m rectangle street area through OpenStreetMap [57], the satellite map of which is presented in Figure 3a. Then, we use Simulation of Urban Mobility (SUMO) [58] to convert the extracted area to the road networks. Figure 3b shows the converted road network of the selected area in Los Angeles. The realistic mobility trace of vehicles is generated by the open-source microscopic space-continuous and time-discrete vehicular traffic generator package SUMO. SUMO uses a collision-free car-following model to determine the speeds and the positions of the vehicles. The output from SUMO is converted into input files for the movement of nodes in the NS-3 simulator. As for the V2V communication, the Nakagami-*m* channel model is applied. We set the value of *m* according to the communication distance. When the distance is less than 50 m, *m* is set as one. When the distance is in the range of [50, 150], *m* is set as 1.5. When the distance is larger than 150 m, *m* is set as two.

The coverage of V2V communications is set as 300 m. The transmission frame duration is set as 1 ms. The average encounter duration is related to the vehicle’s velocity and density. The MAC layer protocol follows 802.11p, with the distributed coordination function enabled. We refer to [38] and set the simulation time as one hour.

We conduct two sets of simulations, which are implemented in the urban and highway environment. We set up different numbers of vehicles to indicate normal traffic conditions and sparse traffic conditions, rather than a static number of vehicles. In each set, the replication limit of message *M* is indicated by parameter *n*; in other words, the number of copies that can be spread is *n*. As for the value of parameter *n*, we apply the method in [11] for calculation. We also vary *n* to see the effect of different numbers of message copies on network performance. Note that in the tradeoff analysis, all metrics are plotted as functions of the number of copies and the number of vehicles, respectively. We repeat the simulations and provide the results plotted with the confidence interval. The main experimental parameters are shown in Table 1.

### 5.2. Performance Metrics

In the simulation, we vary two parameters including the number of message copies and the number of vehicles in the VANET. The range of these parameters will be elaborated along with the performance analysis.

The performance is measured using the following criteria: data delivery ratio, transmissions and dissemination delay. The data delivery ratio is the ratio of the number of vehicles that receive the message to the total number of copies. We use the data delivery ratio to evaluate the dissemination performance of the compared algorithms. Transmissions are denoted by the number of stages (see Definition 5), which characterizes the complexity for the system to enter into a balanced status among a set of nodes. As the number of stages can reflect how many transmissions are needed to achieve an ϵ-balanced status, it is utilized as a measurement of communication overhead to indicate how many average operations the system would perform when it reaches network consensus. We choose data dissemination delay as a measurement of effectiveness, which indicates the time interval from the beginning of data dissemination to the moment that the network enters into a balanced status.

Here, it is worth noting that in the compared schemes, to mitigate the simulation complexity, the number of message copies *n* represents the maximum message copies that can be spread in the process of data dissemination. In replication-based algorithms, such as epidemic routing, SW [35] and capacity-constrained replication algorithm (CCR) [11], *n* is set as the replication limit of the message. As for greedy perimeter stateless routing (GPSR) , a single-copy routing algorithm, we use *n* to denote the the maximum number of nodes that can receive the message, which increases when the node carries a message meeting a node without the message.

### 5.3. Data Delivery Ratio

In this section, we compare the performance of the proposed algorithm EDDA with other data dissemination schemes, such as GPSR, epidemic routing, SW and CCR, in terms of data delivery ratio. We also show how the data delivery ratio is affected by the number of maximum message copies and the number of vehicles. Here, we use the number of vehicles to indicate the network size.

**Figure 4:**
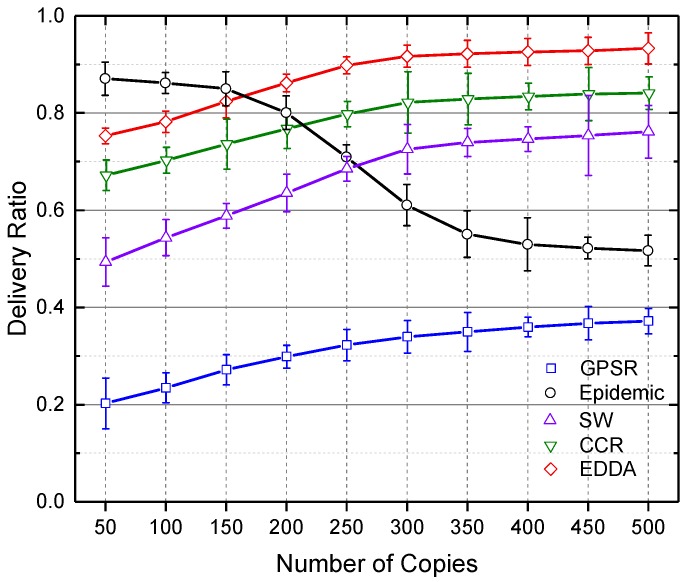
Delivery ratio vs. number of message copies in urban VANET.

**Figure 5:**
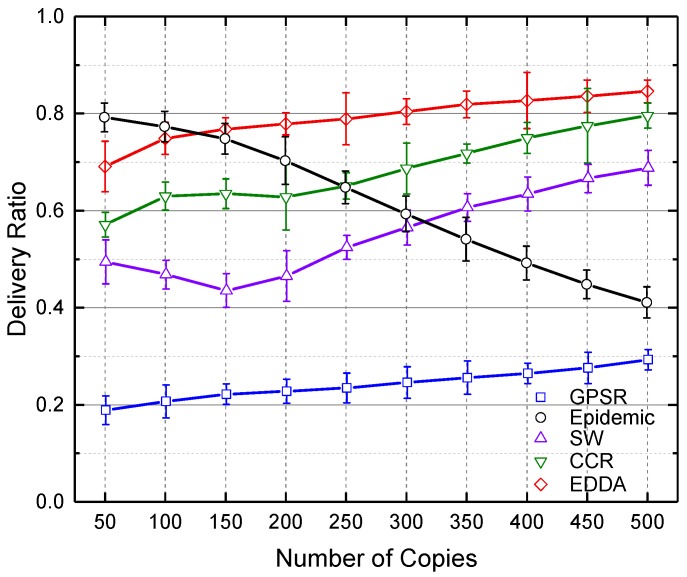
Delivery ratio vs. number of message copies on the highway.

Figure 4 and Figure 5 show the delivery ratio as a function of the number of message copies when the number of vehicles is fixed as 300. It compares data dissemination performance under different numbers of message replicas in the network. Figure 4 shows the results in a regular urban environment, and Figure 5 describes the case of a highway. It is easy to see that when the number of message copies is low, the epidemic algorithm achieves the best delivery ratio. However, as the number of message copies increases in the network, the network traffic dramatically grows in epidemic routing, as well as the number of data collisions increases and the data delivery ratio reduces. As a result, the performance of epidemic routing decreases and gradually underperforms the other algorithms. On the contrary, EDDA outperforms the other compared algorithms when more messages are spread in the network.

**Figure 6:**
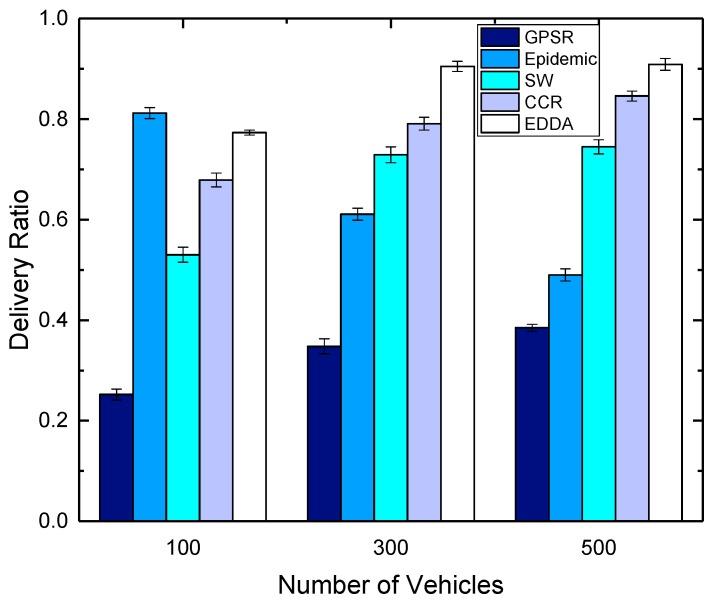
Delivery ratio vs. number of vehicles in urban VANET.

**Figure 7:**
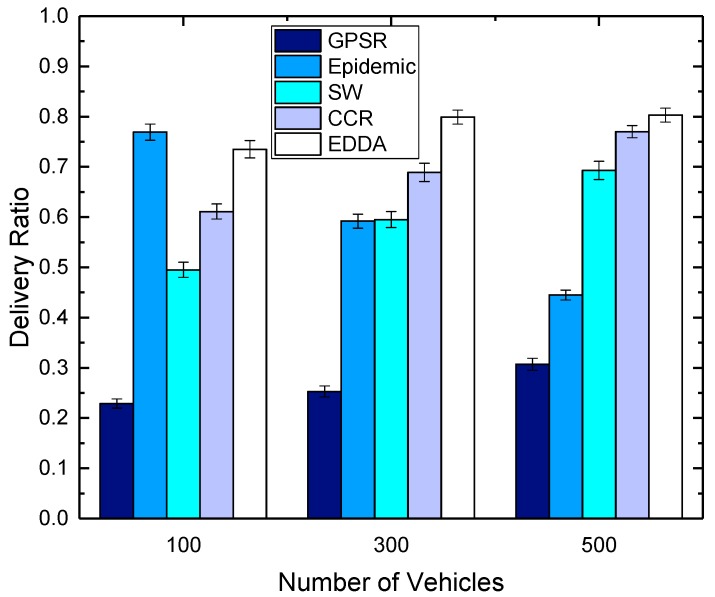
Delivery ratio vs. number of vehicles on the highway.

Figure 6 and Figure 7 show the delivery ratio as a function of the number of vehicles. In the simulations, we fix the maximum message copies that can be replicated as 300, and the number of vehicles is set as 100, 300 and 500. Figure 6 shows the results in urban VANET, while Figure 7 describes the results of highway. We can see that the delivery ratio increases when there are more vehicles in the area. This is because when there are very few vehicles on the roads, it may be hard to find the nearby forwarding vehicles, and hence, messages are dropped after the wait time. When there are more vehicles on the roads, the network connectivity becomes better as the vehicle density is higher, and the frequent node mobility will help carry and forward the packets that temporarily reach the sparse area.

### 5.4. Transmissions

**Figure 8:**
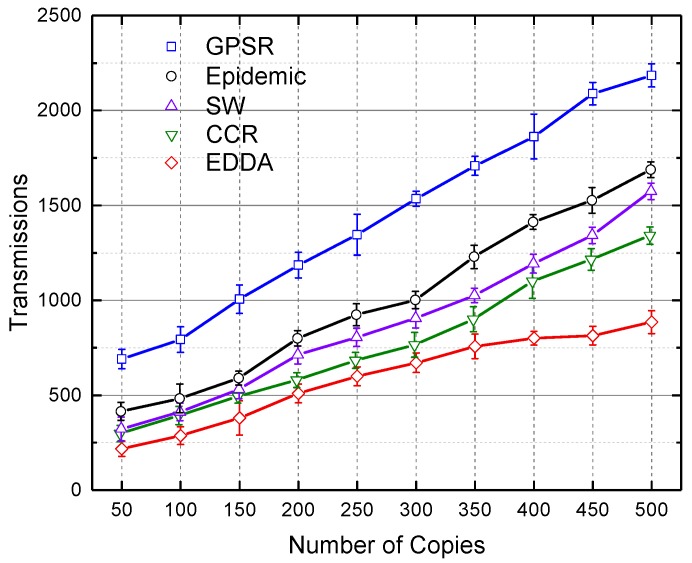
Transmissions vs. number of message copies in urban VANET.

**Figure 9:**
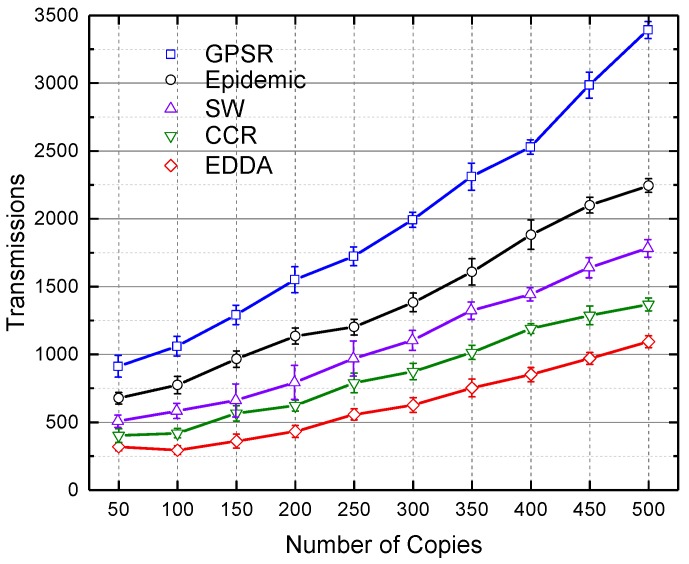
Transmissions vs. number of message copies on the highway.

Figure 8 and Figure 9 respectively depict the total number of transmissions for all compared algorithms when the number of message copies varies from 100–500 under the circumstances of urban and highway. As is evident from these two figures, EDDA performs significantly fewer transmissions than other algorithms. As the number of message copies increases, data transmissions will increase rapidly until the network converges, thus more redundant transmissions would occur. Especially, GPSR and epidemic need more transmissions to achieve ideal data dissemination performance. SW sprays first and then waits for another spray, which results in fewer transmissions. Likewise, CCR can also achieve better performance by taking advantage of the available network capacity. Benefiting from the bounded number of message copies and distributed averaging operations, EDDA outperforms the other compared schemes.

**Figure 10:**
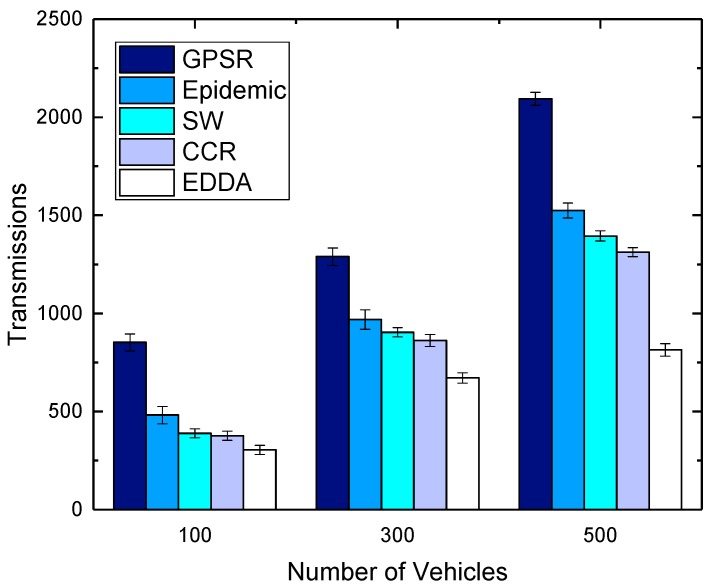
Transmissions vs. number of vehicles in urban VANET.

**Figure 11:**
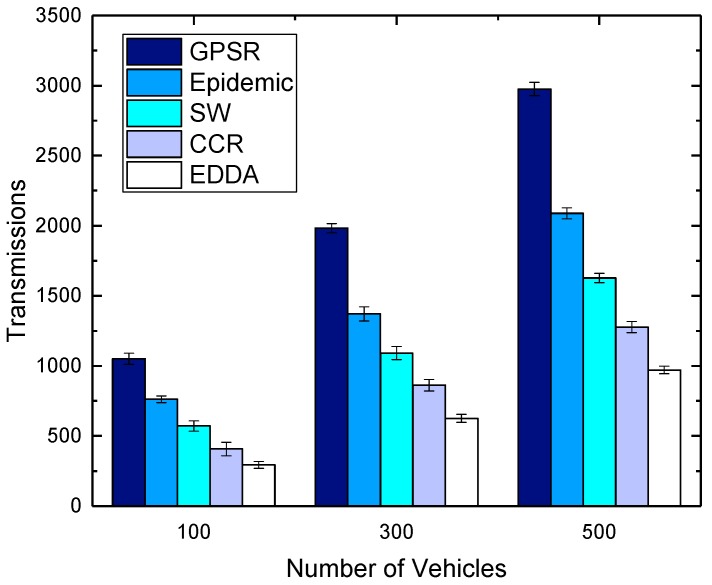
Transmissions vs. number of vehicles on the highway.

Figure 10 and Figure 11 show the impact of the number of vehicles on transmissions for the compared schemes in urban and highway, respectively. It is obvious to see that the number of transmissions increases greatly when the vehicle density is larger. This is because when more vehicles participate in data dissemination, more communications happen among vehicles. However, the trends of the transmission amount for all the compared algorithms are similar. EDDA always consumes fewer transmissions when compared with other algorithms. We can get such a result that the proposed algorithm can achieve better results even in a traffic scenario with lower vehicle density.

### 5.5. Data Dissemination Delay

**Figure 12:**
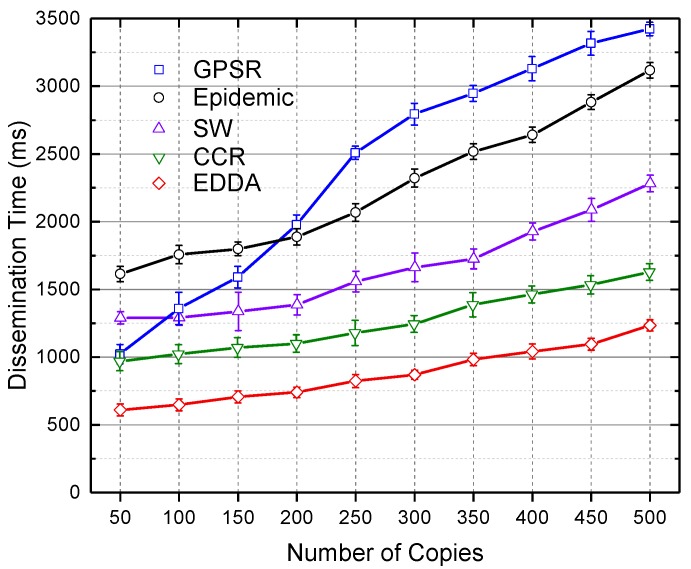
Dissemination delay vs. number of message copies in Urban VANET.

**Figure 13:**
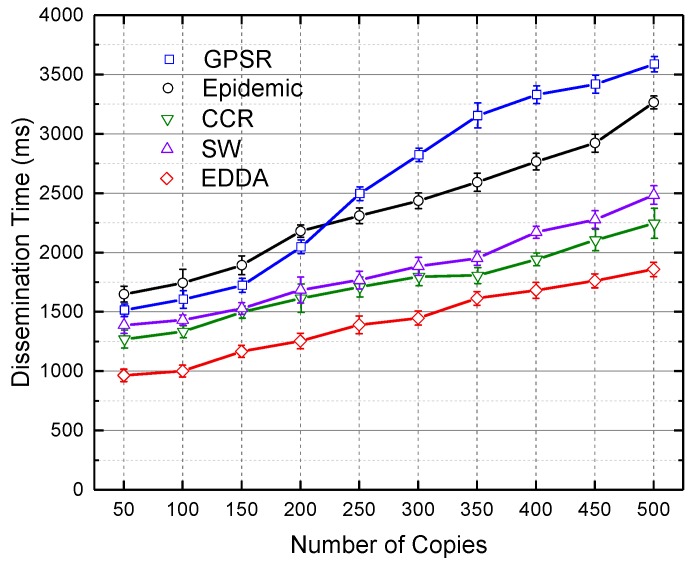
Dissemination delay vs. number of message copies on the highway.

In this section, we compare the data dissemination delay of the compared algorithms. The compared algorithms are GPSR, the epidemic routing scheme, SW and CCR. We will show how data dissemination delay changes when the number of message copies varies from 100–500 in Figure 12 and Figure 13. Figure 12 shows the results in the regular urban environment, and Figure 13 describes the situation of the highway. When the number of message copies increases, the dissemination time increases. The possible reason is that the more messages to be delivered, the more data transmissions occur until the network converges, hence causing the increasing dissemination delay. We again observe that our proposed EDDA achieves better dissemination delay.

**Figure 14:**
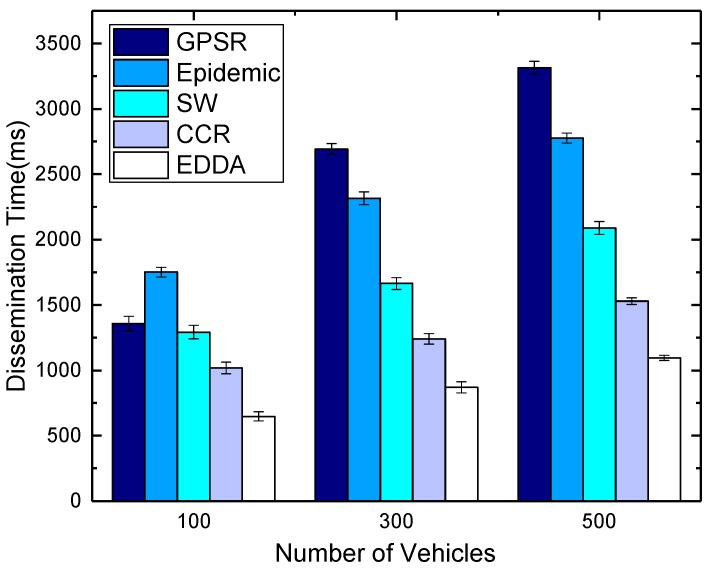
Dissemination delay vs. number of vehicles in urban VANET.

**Figure 15:**
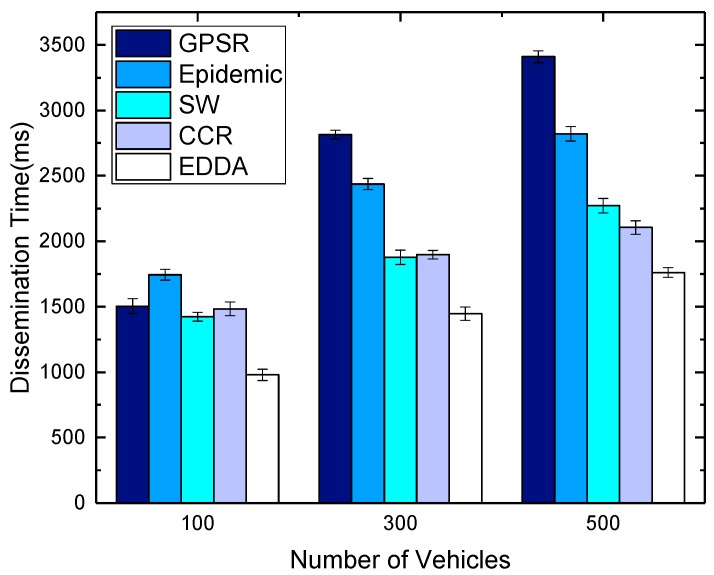
Dissemination delay vs. number of vehicles on the highway.

Figure 14 and Figure 15 compare the effect of different vehicle densities when the number of message copies is equal to 300. From the differences in the figures, we can see that data dissemination delay increases with the increasing vehicle nodes. In the scenarios when the number of vehicles increases, the source vehicle typically needs to make multiple attempts to disseminate the messages to other vehicles in the destination area, which increases the overall dissemination time. Epidemic routing and the rest of the schemes manage to achieve good delays for low vehicle density, but perform poorly for most values. GPSR has a relatively low dissemination delay in the sparse environment, and the delay will increase when the network becomes dense. On the other hand, due to the limitation that the number of message copies that can be spread is bounded, SW, CCR and EDDA exhibit greater stability. They perform fewer transmissions in all scenarios, while achieving slightly increased dissemination delays as the level of network connectivity increases. As is evident from the figures, in terms of dissemination delay, if traffic loads are low, it is known that most of the schemes can obtain good delays under these conditions. Nevertheless, if traffic starts increasing, EDDA actually outperforms all schemes in terms of delay.

### 5.6. Evaluation of Network Balance on the Highway

In this section, we particularly discuss the situation when the algorithm is applied to the highway environment. We will see the differences between the delay for the network to enter into a balanced status and the delay for the network to rebalance if a new node enters the network and breaks the previous balance. Note that we assume vehicles do not take over each other on the highway in order to maintain an order of vehicles and simplify the problem.

Here, we use two parameters $t_{1}$ and $t_{2}$ to indicate the delays. $t_{1}$ indicates the time for the system to enter into balance. Then, we consider the situation that a new vehicle node joins the balanced system. Obviously, the system would need to do additional average operations to be rebalanced. $t_{2}$ is used to indicate the time for the system to be rebalanced.

**Figure 16:**
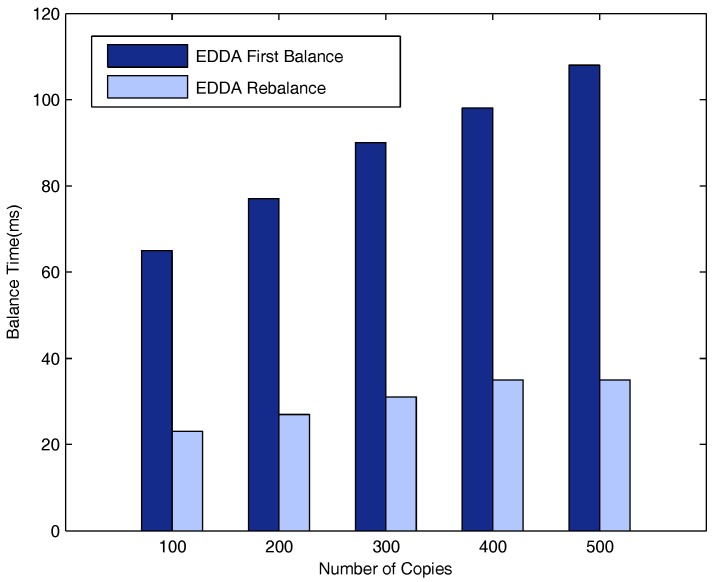
Time vs. number of message copies when the number of vehicle nodes is 50.

**Figure 17:**
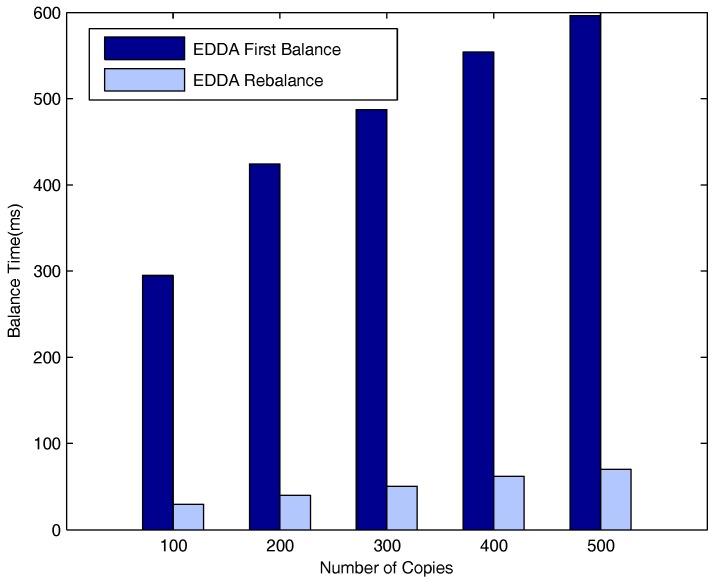
Time vs. number of message copies when the number of vehicle nodes is 100.

**Figure 18:**
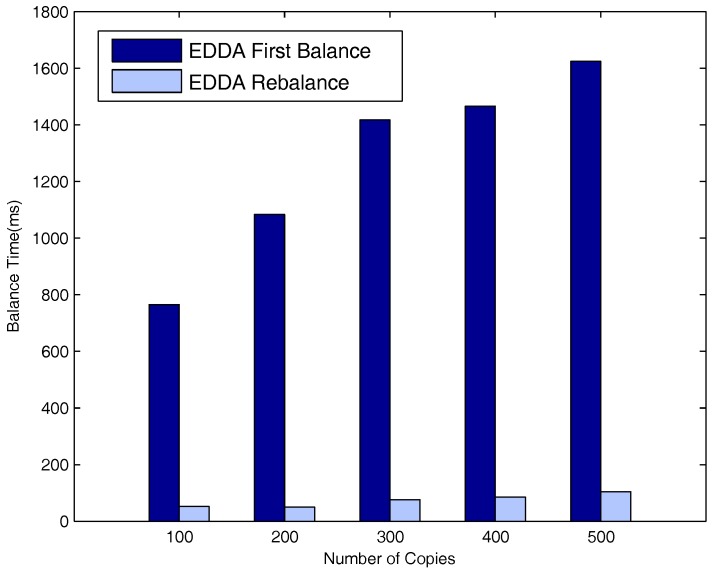
Time vs. number of message copies when the number of vehicle nodes is 150.

Figure 16, Figure 17 and Figure 18 respectively describe the delays for the system to enter into a balanced status when the number of maximum allowed message copies varies from 100–500. The number of vehicle nodes is 50 in Figure 16, 100 in Figure 17 and 150 in Figure 18. As is evident from the three figures, both $t_{1}$ and $t_{2}$ increase when the maximum number of message copies increases from 100–500. Besides, we can easily see that $t_{2}$ is much smaller than $t_{1}$. This is because when new nodes join the network, they will break the system balance, and the system needs to do additional average operations to be balanced again. Nevertheless, it needs fewer average operations to obtain system rebalance than the initial balance status.

**Figure 19:**
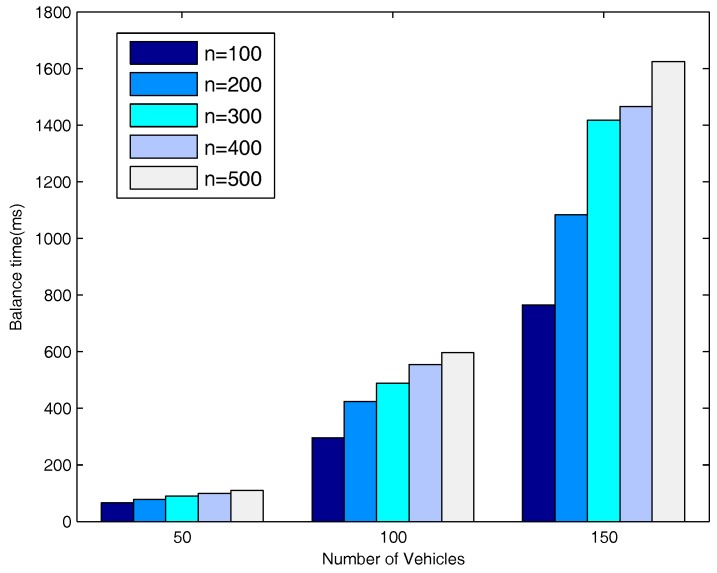
$t_{1}$ VS. number of vehicles.

Figure 19 shows the delays for the system to enter into a balanced status when the number of vehicle nodes varies from 50, to 100, to 150. The figure also provides a comparison of the delays when the number of message copies is 100, 200, 300, 400 and 500. As can be seen from the figure, when the number of message copies is fixed, the time to balance the system increases as the number of vehicle nodes increases since it needs to do more average operations.

## 6. Conclusions

To facilitate data dissemination in VANETs, we investigate distributed data replication algorithms. We use graph theory to describe the network topology and discuss the cases of arbitrary graph and linear graph. Then, we propose a distributed data replication algorithm with a bounded number of message copies disseminated to an area of interest. Moreover, we measure the complexity of network convergence by the number of communication stages in a distributed computing environment. We prove that there are at least $\min(\frac{n}{2+\epsilon },m)$ nodes that can receive the message when the system enters into an ϵ-balanced status. Finally, detailed analysis of the convergence speed of the proposed algorithm, as well as the upper bound and lower bound are given. It shows that through pairwise average operations, the network can enter into a balanced status in a quick manner. Simulations driven by real traces in Los Angeles have been done to demonstrate the effectiveness and the superior performance of the proposed solution, which results in a substantial dissemination delay reduction and decreasing communication overhead compared with the state-of-the-art solutions.

## Figures and Tables

**Table 1:** Parameters used in the simulations.

Parameter	Value
Simulation area	2000 m × 2000 m
Simulation time	1 h
Vehicle communication range	300 m
Vehicle velocity	[30, 120] km/h
Number of Vehicles	100, 300, 500
Number of message copies	[50, 500]

## Abbreviations

The following abbreviations are used in this manuscript:

VANETs	Vehicular ad-hoc networks
V2V	Vehicle-to-vehicle
RSU	Road side unit
EDDA	Efficient distributed data replication algorithm
